# Effects of Neurotrophic Support and Amyloid-Targeted Combined Therapy on Adult Hippocampal Neurogenesis in a Transgenic Model of Alzheimer's Disease

**DOI:** 10.1371/journal.pone.0165393

**Published:** 2016-10-21

**Authors:** Christopher D. Morrone, Lynsie A. M. Thomason, Mary E. Brown, Isabelle Aubert, JoAnne McLaurin

**Affiliations:** 1 Biological Sciences, Sunnybrook Research Institute, M4N 3M5, Toronto, ON, Canada; 2 Department of Laboratory Medicine and Pathobiology, University of Toronto, M5S 1A8, Toronto, ON, Canada; 3 Physical Sciences, Sunnybrook Research Institute, M4N 3M5, Toronto, ON, Canada; Rosalind Franklin University of Medicine and Science, UNITED STATES

## Abstract

Although it is recognized that multi-drug therapies may be necessary to combat AD, there is a paucity of preclinical proof of concept studies. We present a combination treatment paradigm, which temporally affects different aspects of Alzheimer’s disease (AD)-like pathology, specifically Aβ-toxicity and neurogenesis. At early stages of AD-like pathology, in TgCRND8 mice, we found that combating Aβ pathology with *scyllo*-inositol ameliorated deficits in neurogenesis. Older TgCRND8 mice with established amyloid load had decreased progenitor cell proliferation and survival compared to non-transgenic mice, regardless of *scyllo*-inositol treatment. The prolonged exposure to Aβ-pathology leads to deficits in the neurogenic niche, thus targeting Aβ alone is insufficient to rescue neurogenesis. To support the neurogenic niche, we combined *scyllo*-inositol treatment with leteprinim potassium (neotrofin), the latter of which stimulates neurotrophin expression. We show that the combination treatment of *scyllo*-inositol and neotrofin enhances neuronal survival and differentiation. We propose this proof of concept combination therapy of targeting Aβ-pathology and neurotrophin deficits as a potential treatment for AD.

## Introduction

Alzheimer’s disease (AD) is a neurodegenerative disease that most commonly affects the elderly population [1]. The AD brain is characterized by two distinct lesions, Aβ plaques and neurofibrillary tangles [2]. The most advanced therapeutic strategies presently under investigation target the toxic Aβ peptide. However, many have failed to meet primary cognitive endpoints, possibly as a result of intervention after the long prodromal phase of AD that relates directly to Aβ accumulation [3]. In light of this, understanding pathways that are not rescued or stimulated after removal of Aβ may represent novel strategies for combination therapies.

One process that is particularly vulnerable during aging and disease is hippocampal neurogenesis, shown to be altered in both AD patients [4–5] and AD mouse models [6–8]. Previous studies demonstrate that neurogenesis is required for many hippocampal-dependent tasks (reviewed in [9]), in particular for pattern separation [10–11], which is defective in AD patients [12].

Continual insult by Aβ leads to dysregulation of the neurotrophic environment. Notably, levels of brain derived neurotrophic factor (BDNF) are decreased, and levels of pro-nerve growth factor (NGF), which has a high affinity for p75 neurotrophin receptor (p75^NTR^) and the downstream pro-apoptotic pathways, are increased [13–15]. BDNF and NGF promote the growth, survival and differentiation of neurons and are associated with increases in neurogenesis [16–17]. Thus, with disease progression, early stage Aβ-induced neuronal dysfunction might be rescued solely by amelioration of toxic Aβ species. However, at later stages of pathology and loss of trophic support, a multi-targeted therapy may be required to rescue neurogenesis.

We have previously demonstrated that treatment of disease-bearing TgCRND8 mice with the orally available Aβ targeting compound, *scyllo*-inositol, reduced Aβ pathology and improved spatial cognitive function [18–19]. Recently, further preclinical data demonstrated that *scyllo-*inositol reversed deficits associated with the infusion of Aβ oligomers, a model of neuronal injury rather than loss [20]. A phase II clinical trial with *scyllo-*inositol in mild to moderate AD patients demonstrated Aβ target engagement and high *scyllo-*inositol CNS bioavailability, however it did not meet the primary cognitive endpoints [21]. In light of these combined preclinical and clinical data, *scyllo*-inositol was utilized here to modulate Aβ across disease progression. Whereas to address the loss of trophic support, we utilized neotrofin (AIT-082; leteprinim potassium) [22]. Neotrofin is a purine hypoxanthine derivative, which has been shown to increase the levels of NGF, BDNF and other neurotrophins [23–24]. Neotrofin promotes neuritogenesis *in vitro* and protects against age-related memory decline [22–23]. A single dose of neotrofin stimulates hippocampal proliferation in non-transgenic mice [25], suggesting that neotrofin treatment may enhance neurogenesis in AD.

In the present study, we characterized the deficits in neurogenesis as a function of age- and disease-progression in TgCRND8 transgenic (Tg) and non-transgenic (NTg) mice. The reduction of Aβ levels after treatment with *scyllo*-inositol normalized progenitor cell proliferation and enhanced the neuronal survival in Tg mice early in disease but not in advanced disease. Combination treatment with *scyllo*-inositol and neotrofin, at an advanced disease stage in Tg mice, was required to increase both neuronal differentiation and survival. Thus our data support combination therapeutic strategies over the course of AD progression.

## Material and Methods

### Animals and Treatment

**Fig 1:**
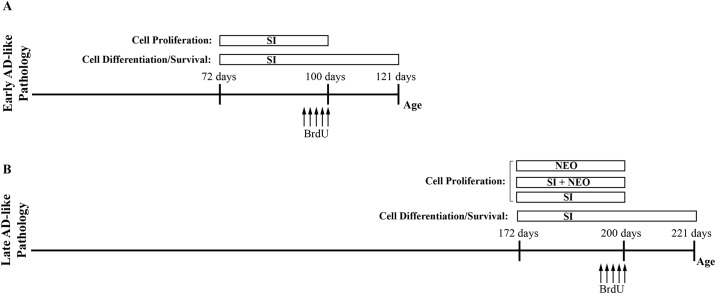
Timeline of therapeutic interventions. (A) TgCRND8 (Tg) mice with early AD-like pathology and age-matched non-transgenic (NTg) mice were treated or untreated from 72 days of age until sacrifice at either 100 or 121 days, for proliferation and differentiation/survival, respectively. BrdU was intraperitoneally injected in these cohorts of mice for 5 days from day 96–100. (B) Tg mice with late AD-like pathology and age-matched NTg mice were treated or untreated from day 172 days of age until sacrifice at either 200 or 221 days. Tg mice were treated with *scyllo*-inositol (Tg-SI), neotrofin (Tg-NEO) or a combination of both (Tg-SI/NEO) for 28 days. BrdU was injected in these cohorts of mice for 5 days from day 196–200.

TgCRND8 mice overexpress human amyloid precursor protein (APP695) containing the Swedish (KM670/671NL) and Indiana (V717F) familial AD mutations under the control of the Syrian hamster prion promoter on an out-bred C57BL6/C3H background [26]. Mice were bred in house and were kept on a 12 hour light-dark cycle with *ad libitum* access to chow and water. All methods were approved by the University of Toronto’s Animal Care Committee, and were performed in accordance with the Canadian Council on Animal Care guidelines. Early/humane endpoints for severely ill animals were based upon standard operating procedures in accordance with the Canadian Council on Animal Care guidelines. The Toronto’s Animal Care Committee approved the following protocols: #20010221 (October 14, 2013–2014); #20009707 (November 11, 2012–2013); #20009120 (November 25, 2011–2012). Sex-balanced and aged-matched Tg and NTg littermate mice were randomly divided into treatment and non-treatment groups (n = 5–7). *scyllo-*Inositol (kind gift of Transition Therapeutics Inc), 10mg/ml, was administered *ad libitum* in drinking water as per [19]. Neotrofin (leteprinim potassium; Santa Cruz) was delivered intraperitoneally (I.P.) at 60mg/kg in sterile saline, three times weekly for 4 weeks (dosing as per [27]). *scyllo*-Inositol and/or neotrofin treatment began at 72 or 172 days of age and continued until sacrifice (Fig 1). Each mouse was administered a daily intraperitoneal injection of 50mg/kg of 5-bromo-2’-deoxyuridine (BrdU) (Sigma-Aldrich) for 5 days, ending at 100 or 200 days of age to label dividing cells (Fig 1). This BrdU paradigm allows labelling of cells that proliferated during the 5 days of injections. At the end of each treatment paradigm, under anesthesia with 120-160mg/kg pentobarbital (I.P.), mice were transcardially perfused with 0.1M phosphate buffered saline (PBS) followed by 4% paraformaldyhyde in 0.1M PBS (pH~7.3) to allow for immunohistochemical analysis of the tissue. Mice were handled 5–7 days prior to the beginning of treatment to reduce the stress of I.P. injections. Mice were monitored regularly throughout the course of the experiment and no mice were lost to attrition in this study.

### Immunofluorescence Staining

Forty-micron coronal sections were taken throughout the hippocampus (Bregma -1.28 mm to -2.92 mm) using a sliding microtome, Microm HM400 (Thermo Scientific). One in 8 sections (320 μm apart), for a total of 7 evenly spaced sections per animal, were selected across the span of the dentate gyrus for immunofluorescence staining. Primary antibodies, polyclonal rabbit anti-glial fibrillary acidic protein (GFAP) (1:500, Dako, Z0334), polyclonal goat anti-doublecortin (1:300, Santa Cruz, sc-8066), monoclonal rat anti-BrdU (1:400, AbD Serotec, OBT0030), monoclonal mouse anti-neuronal nuclear antigen (NeuN) (1:200, Millipore, MAB377) and polyclonal rabbit anti-calretinin (CR) (1:250, Abcam, ab702) were utilized. Briefly, sections were blocked in 1% bovine serum albumin (BSA), 2% donkey serum, 0.1% Triton X-100 in PBS (PBS++) for 1 hour and all antibodies were diluted with PBS++. Primary antibody incubation occurred overnight at 4°C followed by Alexa Fluor-conjugated secondary antibody (1:200, Invitrogen) incubation for 2 hours at room temperature. Antigen retrieval was necessary for BrdU staining using 2N hydrochloric acid (HCl) for 30 minutes at 37°C. To neutralize the HCl, sections were rinsed with 0.1M borate buffer (pH 8.5) for 10 minutes. All immunofluorescence was analyzed on a Leica TCS SP5 confocal microscope at 40x (100/121/200/221 day old NTg, NTg-SI, Tg and Tg-SI) or 20x (200 day old Tg-SI, Tg-NEO, and Tg-SI/NEO) magnification. Z-stacks were collected and cells were counted throughout the x, y and z plane of the sections.

Sampling for neurogenesis was done in accordance with [28], with the goal of counting approximately 100 cells from evenly spaced sections across the hippocampus to estimate the total number of cells across the dentate gyrus. In the young 100 day old animals, with high levels of cell proliferation, 3 fields of view in the dentate gyrus per section (covering approximately 30% of the right dentate gyrus), within the right hemisphere were systematically chosen for imaging and analysis. The number of BrdU+ cells per entire dentate gyrus was extrapolated by multiplying the number of BrdU+ cells counted by the ratio of dentate gyrus area counted to the total dentate gyrus area across the 7 sections. This was then multiplied by 8 to achieve an estimate of the number of BrdU+ cells across the entire dentate gyrus. Since the number of BrdU+ cells that survive for approximately 3 weeks is much lower than the number of proliferating cells, and because there are lower endogenous levels of proliferation and survival in the older mice, a more exhaustive counting paradigm was used in the 121, 200 and 221 day old mice. To meet the criteria of counting at least 100 cells in these mice,[28] all cells in the dentate gyrus of both hemispheres were counted for the 7 sections per animal, and this number was multiplied by 8 to estimate the total number of cells across the entire dentate gyrus. These counting protocols were used for all cell types in which a total count was determined: BrdU+ cells at all ages, and DCX+ and CR+ cells in the 200 day old mice. No further sampling was required because we analyzed every cell within the total counts for colocalization with BrdU (nucleus), NeuN (nucleus), GFAP (cytoplasm), DCX (cytoplasm), and/or CR (cytoplasm and nucleus), using Z-stacks to confirm co-labelling in the x, y and z planes. Only cells present in the subgranular zone and the granular cell layer were analyzed. Colocalization required overlapping staining in the x,y and z planes throughout the Z-stacks. Cells that did not meet these criteria were excluded from colocalization and counted as single labelled cells. Dentate gyrus images were stitched together in ImageJ as per [29].

### Aβ Plaque Immunohistochemical Staining

To assess hippocampal Aβ plaque load immunohistochemical analyses were performed as previously reported [18]. For the 121 day old mice 1 in 8 sections (for a total of 7 sections/animal) were selected and for the 200 and 221 day old mice 1 in 14 sections (for a total of 4 sections/animal) were selected. Briefly, endogenous peroxidases were blocked with 1% hydrogen peroxide for 30 minutes, followed by antigen retrieval with 70% formic acid for 5 minutes. Sections were incubated overnight with mouse anti-human amyloid-beta peptide (6F/3D) antibody (1:400; Dako) in 0.2% BSA, 0.2% Triton X-100 in PBS. Sections were then incubated with a Vectastain ABC kit (Vector Laboratories) anti-mouse secondary antibody (1:200) for 1.5 hours, followed by the Vectastain ABC solution for 1 hour. Sections were developed with a DAB peroxidase substrate kit (Vector Laboratories). Images of the right and left hippocampus were obtained on a Leica DMI3000 Invertfluor scope (Leica). ImageJ was used to measure the hippocampal area, plaque number and size. Images were scaled, processed to remove background and binarized before plaque number and size were assessed. Percent area covered in plaques was calculated by dividing the total area of plaques by the total hippocampal area measured per mouse.

### Statistical Analyses

Statview (SAS Institute Inc.) or GraphPad Prism 6 (GraphPad Software, Inc.) was used for statistical analyses. One-way analysis of variance (ANOVA) with a Fisher’s Post-hoc test was used for multiple comparisons. Unpaired t-test was used for comparisons involving 2 groups. All data are presented as the mean ± standard error of the mean (SEM). The data for all figures can be found in supplemental data.

## Results

We studied AD-related neurogenesis in the TgCRND8 (Tg) mice at stages modelling early and advanced Aβ-pathology. We investigated the effect of the reduction of Aβ-pathology by *scyllo-*inositol treatment on neurogenesis in mice with early (100/121 days old, Fig 1A) and late (200/221 days old, Fig 1B) stage pathology. In late AD-like pathology (200 days old) we also combined *scyllo*-inositol with neotrofin to determine if this combination treatment had a beneficial effect on neurogenesis (Fig 1B). No sex differences were detected in any of the following results.

### Cell proliferation, differentiation and survival in early AD-like Pathology

**Fig 2:**
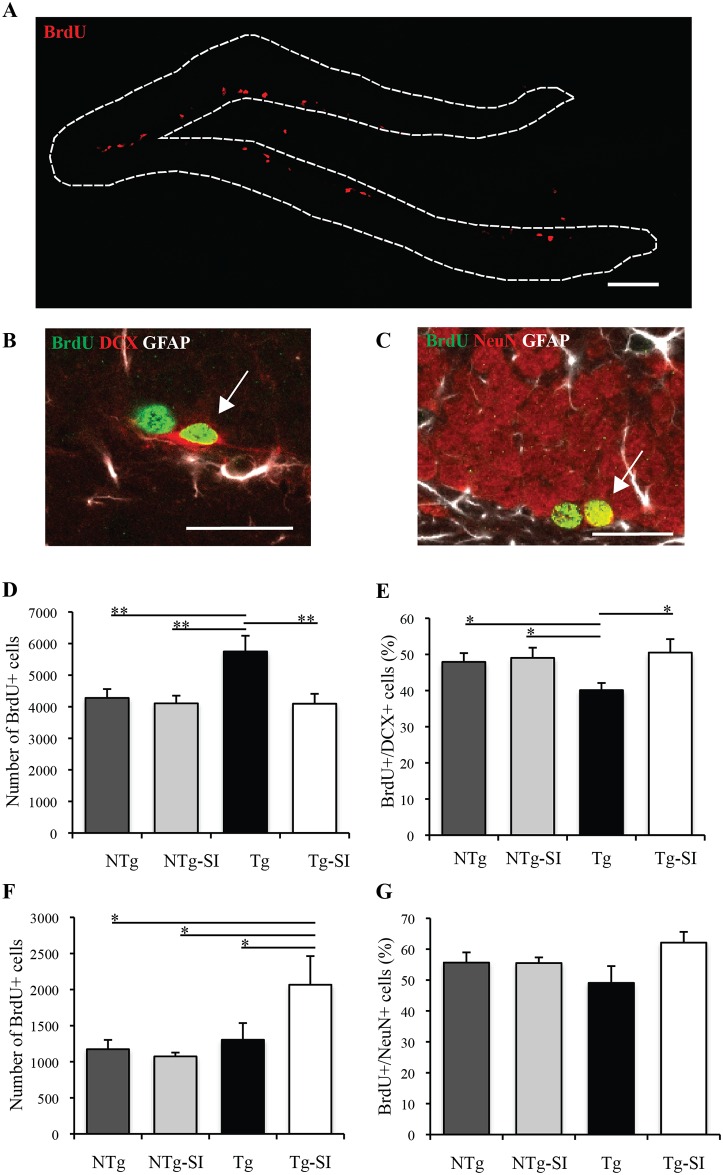
Hippocampal cell proliferation, differentiation and survival in Tg mice with early AD-like pathology and age-matched Tg littermates. (A) A representative image of the dentate gyrus stained for BrdU, demonstrates the distribution of proliferating BrdU+ cells. (B) A representative image of BrdU (green), DCX (red) and GFAP (white) positive cells in the dentate gyrus at 100 days of age. The arrow highlights a representative BrdU+/DCX+ cell. (C) A representative image of BrdU (green), NeuN (red) and GFAP (white) positive cells in the dentate gyrus. The arrow highlights a representative BrdU+/NeuN+ cell. Cell proliferation was examined at 100 days of age (D,E) while differentiation and survival was examined at 121 days of age (F,G) as a function of *scyllo*-inositol treatment. (D) The number of proliferating BrdU+ cells in the dentate gyrus of NTg (n = 7), NTg-SI (n = 6), Tg (n = 7) and Tg-SI (n = 5) mice were compared. This demonstrates more BrdU+ cells in Tg compared to NTg and to Tg-SI. (E) The percentage of BrdU+/DCX+ cells in the dentate gyrus showed less neuronal BrdU+ cells in Tg compared to the other three cohorts. (F) The number of BrdU+ cells surviving in NTg (n = 6), NTg-SI (n = 5), Tg (n = 6) and Tg-SI (n = 6) mice demonstrates more cell survival in Tg-SI mice. (G) The percentage of BrdU+/NeuN+ cells shows no difference between cohorts. Scale bars indicate 100 μm (A) or 25 μm (B,C). Data are mean ± SEM. One-way ANOVA with Fisher’s Post-hoc test, * represents p< 0.05 and ** represents p< 0.01.

Neural progenitor cell (NPC) proliferation within the dentate gyrus was assessed in untreated and *scyllo*-inositol treated Tg and NTg littermate mice at 100 days of age. A representative image of the dentate gyrus stained with BrdU demonstrates the distribution of BrdU+ proliferating cells (Fig 2A). Triple staining was performed to assess whether proliferating cells (BrdU+) were neuronal (DCX+) or GFAP+, and demonstrate colocalized BrdU+/DCX+ neurons (Fig 2B). Triple staining of BrdU/NeuN/GFAP demonstrates colocalized BrdU+/NeuN+ neurons within the dentate gyrus (Fig 2C). A significant effect of genotype and *scyllo*-inositol treatment on the number of BrdU+ cells was detected (F (3, 21) = 5.06, p = 0.009; Fig 2D). Untreated Tg mice have significantly more BrdU+ cells compared to both untreated and treated NTg groups (p = 0.007; p = 0.004, respectively) (Fig 2D). Interestingly, Tg mice treated with 4 weeks of *scyllo*-inositol had less BrdU+ cells (p = 0.006) than untreated Tg mice, and comparable levels to those in untreated and treated NTg mice (Fig 2D). *scyllo*-Inositol treatment had no effect on the number of BrdU+ cells in the NTg mice (p = 0.74; Fig 2D). A significant effect of genotype and treatment was also detected on the percent of proliferating cells that were neuroblasts or immature neurons, DCX+, (F (3, 21) = 3.13, p = 0.047; Fig 2E). Untreated Tg mice showed a lower percentage of BrdU+/DCX+ cells in the dentate gyrus compared to untreated and treated NTg groups (p = 0.04, p = 0.03, respectively) (Fig 2E). *scyllo*-Inositol treated Tg mice had a higher percentage of neuroblasts and immature neurons than untreated Tg mice (p = 0.015), and comparable levels to those observed in untreated and treated NTg mice (Fig 2E). *scyllo*-Inositol had no effect on the percentage of BrdU+/DCX+ cells in the NTg mice (p = 0.77; Fig 2B). Genotype or treatment did not have an effect on the percent of proliferating cells that were GFAP+ (NTg mean = 1.96±0.54; NTg-SI mean = 4.25±1.23; Tg mean = 4.15±0.81; Tg-SI mean = 3.97±1.08; F (3, 21) = 1.551, p = 0.23).

To determine whether *scyllo*-inositol-treatment results in enhanced neuronal differentiation and survival, cells were assessed 3 weeks after the last BrdU injection in the 121 day old mice. Triple staining immunofluoresence determined whether the proliferating cells differentiated into mature neurons (NeuN+) or astrocytes (GFAP+). There was an overall effect of genotype and treatment on the number of surviving BrdU+ cells (F (3, 19) = 3.26, p = 0.04; Fig 2F). The number of BrdU+ cells within the dentate gyrus in Tg mice was not significantly different from untreated and treated NTg mice (p = 0.71; p = 0.54, respectively; Fig 2F). *scyllo*-Inositol treatment led to a significantly higher number of surviving BrdU+ cells compared to untreated Tg mice (p = 0.04) and to untreated and treated NTg groups (p = 0.02; p = 0.01, respectively). Survival was increased by ~50% after *scyllo-*inositol treatment (Fig 2F). There was no overall effect of genotype and treatment on the percentage of BrdU+/NeuN+ cells (F (3, 19) = 1.97, p = 0.15; Fig 2G). A significant effect of genotype and treatment on the percent of BrdU+ cells that were GFAP+ was detected (NTg mean = 1.63±0.44; NTg-SI mean = 2.17±0.90; Tg mean = 6.25±1.68; Tg-SI mean = 4.71± 0.94; F (3, 19) = 3.91, p = 0.025). The Tg mice had a significantly greater percent BrdU+/GFAP+ cells compared to the untreated and treated NTg groups (p = 0.007; p = 0.02, respectively). *scyllo*-Inositol treatment had no effect on the percent BrdU+/GFAP+ cells in the NTg (p = 0.74) and Tg (p = 0.33) mice.

In summary, in early AD-like pathology Tg mice have altered cell proliferation and DCX expression compared to NTg mice. Tg mice also had impaired cell survival as the increased cell proliferation did not lead to an increase in surviving cells. *scyllo*-Inositol treatment of Tg mice with early AD-like pathology ameliorates alterations in neurogenesis with a robust effect on neuronal cell survival.

### Cell proliferation, differentiation and survival in late AD-like pathology

**Fig 3:**
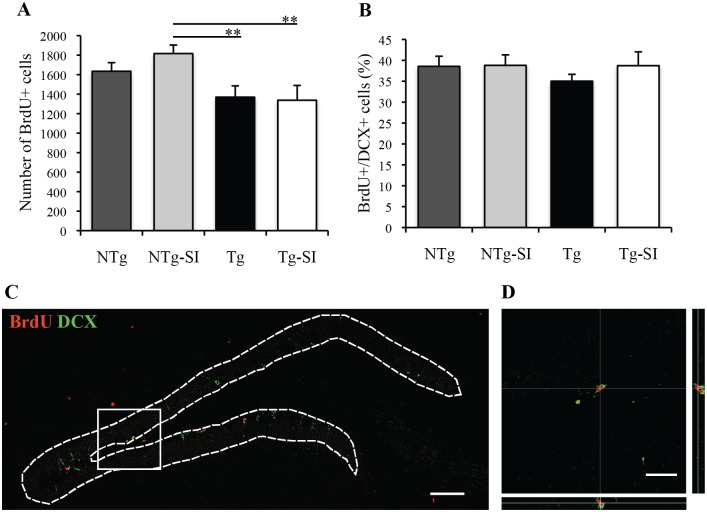
Hippocampal cell proliferation in Tg mice with late AD-like pathology and age-matched NTg littermates. Cell proliferation was assessed at 200 days of age (A,B). (A) The number of proliferating BrdU+ cells in NTg (n = 6), NTg-SI (n = 6), Tg (n = 6) and Tg-SI (n = 5) mice was compared, demonstrating less proliferation in Tg and Tg-SI vs. NTg-SI. (B) The percentage of BrdU+/DCX+ cells in the dentate gyrus was not different between cohorts. (C) Dentate gyrus stained with BrdU (red) and DCX (green) demonstrating the distribution of proliferating cells, as well as neuroblasts and immature neurons, respectively. (D) Representative orthogonal projection of a BrdU+/DCX+ cell. Scale bar indicates 100 μm (C) or 25 μm (D). Data are mean ± SEM. One-way ANOVA with Fisher’s Post-hoc test, ** represents p< 0.01.

NPC proliferation in the dentate gyrus was assessed in untreated and *scyllo*-inositol treated Tg and NTg mice at 200 days of age representing late AD-like pathology (Fig 3). A significant overall effect of genotype and treatment on the number of BrdU+ cells was determined (F (3, 19) = 4.23, p = 0.02; Fig 3A). Tg mice have significantly fewer BrdU+ cells in the dentate gyrus than treated NTg group (p = 0.009; Fig 3A). *scyllo*-Inositol treatment does not rescue this, as *scyllo*-inositol treated Tg mice have a significantly lower number of BrdU+ cells compared to treated NTg group (p = 0.008; Fig 3A). *scyllo*-Inositol treatment had no effect on the number of BrdU+ cells in NTg mice (p = 0.25) or Tg mice (p = 0.85; Fig 3A). There was no effect of genotype or treatment on the percent of BrdU+/DCX+ cells, (F (3, 19) = 0.57, P = 0.64; Fig 3B) or BrdU+/GFAP+ cells (NTg mean = 1.52± 0.39; NTg-SI mean = 1.73±0.62; Tg mean = 2.89±0.76; Tg-SI mean = 2.66±1.15; F (3, 19) = 0.84, P = 0.49). The dentate gyrus stained with BrdU and DCX (Fig 3C), and a co-labelled BrdU+/DCX+ cell (Fig 3D), demonstrate the distribution of BrdU+ proliferating cells and DCX+ cells (neuroblasts and immature neurons).

**Fig 4:**
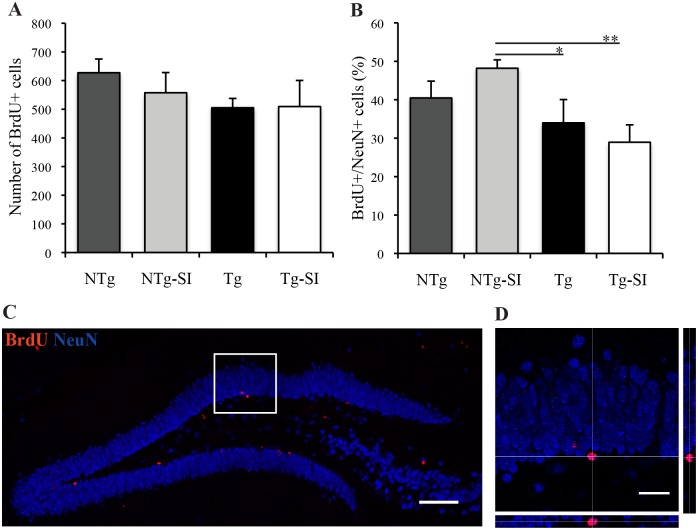
Hippocampal cell differentiation and survival in Tg mice with late AD-like pathology and age-matched NTg littermates. Differentiation and survival was examined at 221 days of age (A,B) as a function of *scyllo*-inositol treatment. (A) The number of BrdU+ cells surviving in NTg (n = 7), NTg-SI (n = 7), Tg (n = 6) and Tg-SI (n = 6) mice showed no difference between treatment or genotype. (B) The percentage of BrdU+/NeuN+ cells in the dentate gyrus showed Tg and Tg-SI mice had a lower percentage of BrdU+/NeuN+ cells compared to NTg-SI mice. (C) Dentate gyrus stained with BrdU (red) and NeuN (blue) demonstrating the distribution of surviving newborn cells (red) within the population of mature granular neurons (blue). (D) Representative orthogonal projection of BrdU+/NeuN+ cell. Scale bar indicates 100 μm (C) or 25 μm (D). Data are mean ± SEM. One-way ANOVA with Fisher’s Post-hoc test, * represents p< 0.05 and ** represents p< 0.01.

NPC differentiation and survival was assessed in untreated and *scyllo*-inositol treated Tg and NTg mice at 221 days of age, to determine the phenotype of surviving BrdU+ cells. There was no significant effect of genotype or treatment on the number of BrdU+ cells (F (3, 22) = 0.8, p = 0.51; Fig 4A). Genotype and treatment had a significant overall effect on the percent of BrdU+/NeuN+ (F (3, 22) = 3.67, p = 0.03; Fig 4B). The percent of newborn mature neurons in treated Tg mice was significantly lower than in treated NTg mice (p = 0.005; Fig 4B). Furthermore, *scyllo*-inositol treatment of the 221 day old animals had no effect on neuronal differentiation in Tg mice (p = 0.44) or NTg mice (p = 0.21; Fig 4B). Genotype and treatment also had a significant overall effect on the percent of BrdU+/GFAP+ cells (NTg mean = 3.67±0.43; NTg-SI mean = 2.30±0.66; Tg mean = 6.l6±1.38; Tg-SI mean = 7.43±1.29; F (3, 22) = 5.81, p = 0.004). Tg mice had a significantly greater percent BrdU+/GFAP+ cells compared to the treated NTg group (p = 0.01). Treated Tg mice also had a significantly greater percent BrdU+/GFAP+ cells compared to the untreated and treated NTg mice (p = 0.01 and p = 0.001, respectively). *scyllo*-Inositol treatment had no effect on the percent BrdU+/GFAP+ cells in the NTg (p = 0.31) and Tg (p = 0.38) mice. The dentate gyrus stained with BrdU and NeuN (Fig 4C), and a co-labelled BrdU+/NeuN+ cell (Fig 4D), demonstrate the distribution of adult-born BrdU+ surviving cells within the granular cell layer, as well as a newborn granule cell. The phenotypic distribution of the surviving BrdU+ cells in 221-day old Tg mice, regardless of treatment, have lower numbers of surviving cells that are of neuronal lineage.

Our data demonstrate alterations in hippocampal neurogenesis in both early and late AD-like pathology in Tg mice. Targeting Aβ removal early in Tg mice rescues these alterations, however in late AD-like pathology does not. It should be noted that *scyllo*-inositol treatment of NTg mice had no effect on cell proliferation, differentiation or survival, suggesting that *scyllo*-inositol does not directly affect progenitor cells.

**Fig 5:**
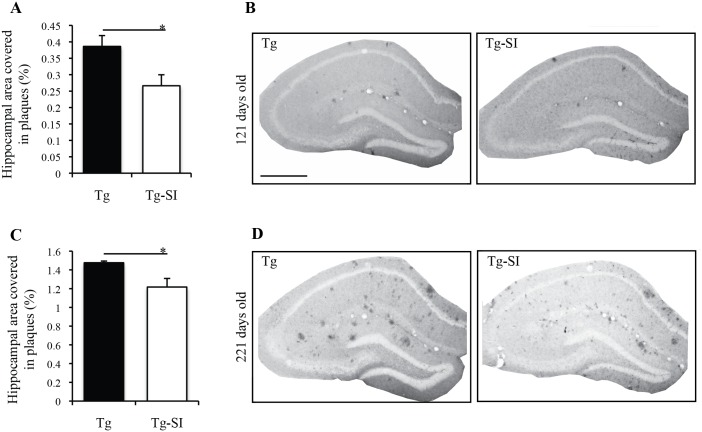
Hippocampal Aβ plaques in 121 and 221 day old *scyllo*-inositol treated and untreated Tg mice. (A) Percent hippocampal area covered in plaques in 121 day old Tg (n = 5) and Tg-SI (n = 6) mice showed decreased plaques after treatment. (B) Representative images of Aβ plaques in the hippocampus of 121 day old mice demonstrate the reduction in plaques after *scyllo*-inositol treatment. (C) Percent hippocampal area covered in plaques in 221 day old Tg (n = 5) and Tg-SI (n = 6) mice also showed less plaques after treatment. (D) Representative images of Aβ plaques in the hippocampus of 221 day old mice demonstrate the reduction in plaques after *scyllo*-inositol treatment. Scale bar indicates 200 μm. Data are mean ± SEM. Unpaired t-test, * represents p< 0.05.

Since *scyllo*-Inositol treatment of Tg mice has previously been shown to reduce Aβ pathology globally in the brain [18], it was necessary to confirm the level of plaque reduction within the hippocampus. The percent hippocampal area covered in plaques in the old Tg mice was 3 times greater than in young mice [26]. *scyllo-*Inositol significantly reduced the percent hippocampal area covered in plaques by 31% in 121 day old Tg mice (p = 0.03; Fig 5A and 5B) and by 18% in 221 day old Tg mice (p = 0.03; Fig 5C and 5D). Small amounts of plaque accumulated near the subgranular zone of the dentate gyrus and in the hilus. The majority of the plaque within the dentate gyrus was in the molecular layer. Importantly, these results demonstrate a reduction of Aβ burden by *scyllo*-inositol in our region of interest.

### Cell proliferation, differentiation and survival in late AD-like pathology after combination treatment

**Fig 6:**
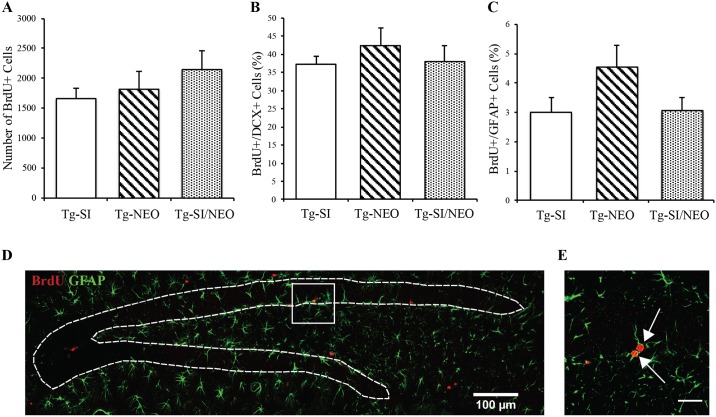
Hippocampal cell proliferation in *scyllo*-inositol, neotrofin, and *scyllo*-inositol/neotrofin treated 200 day old Tg mice. (A) The number of proliferating BrdU+ cells in Tg-SI (n = 7), Tg-NEO (n = 6) and Tg-SI/NEO (n = 7) mice demonstrated no difference between cohorts. (B) The percentage of BrdU+/DCX+ cells (Tg-SI (n = 5), Tg-NEO (n = 6) and Tg-SI/NEO (n = 7)]) and (C) BrdU+/GFAP+ (Tg-SI (n = 7), Tg-NEO (n = 6) and Tg-SI/NEO (n = 7)) in the dentate gyrus of all groups were assessed and showed no changes between treatment groups. (D) Representative image of the dentate gyrus stained with BrdU (red) and GFAP (green) demonstrate the distribution of BrdU+ proliferating cells and astrocytes. (E) Representative BrdU+/GFAP+ cells are indicated by arrows. Scale bar indicates 100 μm (D) or 25 μm (E). Data are mean ± SEM. One-way ANOVA with Fisher’s Post-hoc test.

Since Aβ removal alone did not rescue neurogenesis in late AD-Tg mice, we propose that enhancing neurotrophin support in combination with removal of toxic Aβ may represent a beneficial approach. Neotrofin (AIT-082; leteprinim potassium) was utilized to enhance the expression of neurotrophins as an attempt to enhance neurogenesis, because it was previously shown to effect neurogenesis in non-transgenic mice [25,30]. Furthermore, the neurotrophin imbalance (decreased BDNF levels) are more pronounced in a late-stage compared to an early AD-like pathology in TgCRND8 mice [13], suggesting the need for the combination treatment of neurotrophin enhancement and removal of Aβ in late AD-like pathology. NPC proliferation was assessed as a function of *scyllo*-inositol, neotrofin or *scyllo-*inositol/neotrofin treatment in Tg mice at 200 days of age (Fig 6). No effect of any treatment was detected on the number of BrdU+ cells (F (2, 17) = 0.99, p = 0.39; Fig 6A). The percent of BrdU+ cells that were of immature neuronal lineage (DCX+) was also unchanged across the three treatment groups (F (2, 15) = 0.39, p = 0.68; Fig 6B). There was no significant effect of treatment on percentage of proliferating (BrdU+) cells that were GFAP+ (F (2, 17) = 2.54 p = 0.11; Fig 6C). The dentate gyrus stained with BrdU and GFAP (Fig 6D), and co-labelled BrdU+/GFAP+ cells (Fig 6E), demonstrate the distribution of BrdU+ proliferating cells and astrocytes, as well as newborn GFAP+ cells.

**Fig 7:**
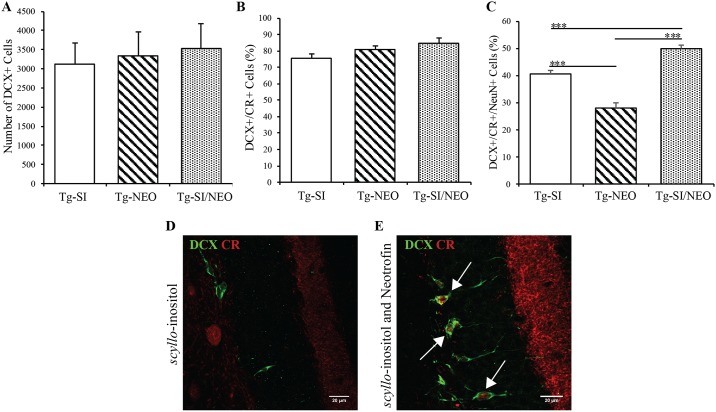
Hippocampal cell differentiation and survival in *scyllo*-inositol, neotrofin, and *scyllo*-inositol/neotrofin treated 200 day old Tg mice. (A) The number of DCX+ cells in Tg-SI (n = 6), Tg-NEO (n = 6) and Tg-SI/NEO (n = 7) mice were not significantly different between cohorts. (B) The percentage of DCX+ cells that were DCX+/CR+ immature neurons were assessed and showed no difference between treatment groups. (C) The percentage of DCX+/CR+ cells that were NeuN+ represents immature neurons approaching maturity. Tg-SI/NEO mice had a significantly greater percentage of DCX+/CR+/NeuN+ cells than Tg-SI and Tg-NEO mice. Tg-SI mice had a greater percentage than Tg-NEO mice. (D/E) Representative images of DCX (green) and CR (red) positive cells in the hippocampus, showing DCX+/CR- and DCX+/CR+ cells in Tg-SI mice and in Tg-SI/NEO mice. (E) Arrows indicate DCX+/CR+ cells. Scale bar indicates 20 μm. Data are mean ± SEM. One-way ANOVA with Fisher’s Post-hoc test, *** represents p<0.001.

To assess neuronal differentiation and survival in 200 day old Tg mice, we examined total number of DCX+ cells and their phenotypes, which includes neuroblasts and immature neurons. The total number of DCX+ cells was not significantly different across all treatments (F (2, 16) = 0.11, p = 0.9; Fig 7A). To distinguish cells differentiating/surviving to an immature neuron phenotype, DCX+/ calretinin+ (CR+) cells were examined. There was no overall effect of treatment on the percent of DCX+ cells that were CR+ (F (2, 16) = 3.01, p = 0.08; Fig 7B). Representative staining of DCX and CR is demonstrated in Fig 7D/7E, showing the DCX+ cells that were CR+ in *scyllo*-inositol treated mice (Fig 7D) and in *scyllo*-inositol/neotrofin treated mice (Fig 7E). The DCX+/CR- cells were observed in, or close to, the subgranular zone, whereas DCX+/CR+ cells migrated into the granular cell layer and exhibited longer processes extending towards the molecular layer of the dentate gyrus (Fig 7D and 7E). Axons within the supramolecular layer, as well as axons and interneurons in the hilus stain for CR, and were similar across all treatment groups (Fig 7D and 7E).

To further delineate the lineage of differentiating/surviving neurons in the dentate gyrus, the DCX+/CR+ cell population was analyzed for co-expression of NeuN. There was an overall effect of treatment on the percentage of DCX+/CR+/NeuN+ cells (F (2, 16) = 54.48, p<0.0001; Fig 7C). *scyllo*-Inositol/neotrofin combination treated mice had a significantly higher percentage of DCX+/CR+ neurons that were also NeuN+, in comparison to *scyllo*-inositol (p = 0.0004) and neotrofin (p<0.0001). s*cyllo-*Inositol treated mice also had a significantly higher percentage of DCX+/CR+/NeuN+ cells than the neotrofin treated mice (p<0.0001; Fig 7C). These data demonstrate that, *scyllo-*inositol/neotrofin combined treatment promotes enhanced differentiation and survival of neuroblasts and immature neurons, and supports this through neuronal maturation (NeuN expression).

**Fig 8:**
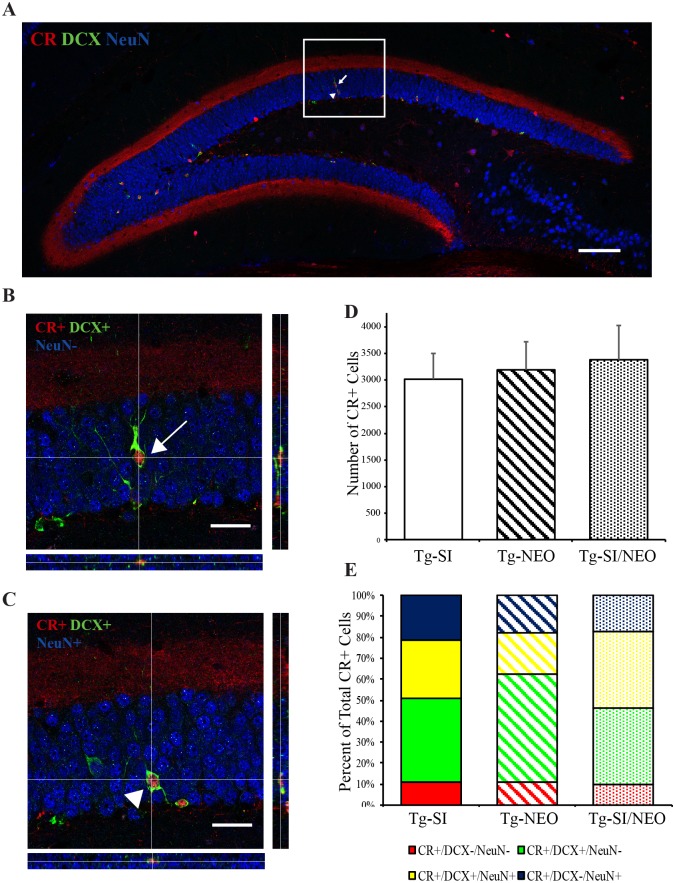
Phenotypic analysis of CR+ cells within the granular cell layer and subgranular zone of the dentate gyrus in 200 day old Tg mice treated with *scyllo*-inositol, neotrofin and *scyllo*-inositol/neotrofin combination. (A) Representative image of the dentate gyrus stained for CR (red), DCX (green) and NeuN (blue). Arrow indicates a CR+/DCX+/NeuN- cell. Arrowhead indicates a CR+/DCX+/NeuN+ cell. (B) Orthogonal projection of CR+/DCX+/NeuN- immature neurons. (C) Orthogonal projection CR+/DCX+/NeuN+ immature neurons. (D) The number of CR+ cells in Tg-SI (n = 6), Tg-NEO (n = 6) and Tg-SI/NEO (n = 7) mice demonstrated no differences between cohorts. (E) Graphical representation of the changes in the percentage of CR+/DCX-/NeuN-, CR+/DCX+/NeuN-, CR+/DCX+/NeuN+ and CR+/DCX-/NeuN+ out of total CR+ cells in Tg-SI, Tg-NEO and Tg-SI/NEO mice. Scale bar indicates 100 μm (A) or 20 μm (B/C). Data are mean ± SEM (D) and mean (E). One-way ANOVA with Fisher’s Post-hoc test.

As immature neurons continue to mature, the expression of DCX is downregulated and thus the DCX-/CR+/NeuN+ immature neuronal population is not captured within the DCX cell population examined in Fig 7. To capture this population, we examined the treatment effect within the total immature neuronal population by phenotyping total CR+ cells in the 200 day old Tg mice treated with either *scyllo*-inositol, neotrofin or *scyllo*-inositol/neotrofin combination (Fig 8). CR+ newborn immature neurons can be separated into CR+/DCX+/NeuN-, CR+/DCX-/NeuN- cells, CR+/DCX+/NeuN+ and CR+/DCX-/NeuN+ cells (Fig 8A, 8B and 8C). The CR+/DCX-/NeuN- and CR+/DCX-/NeuN+ cell populations also include interneurons that could be differentiated morphologically. We demonstrate that the total number of CR+ cells (all phenotypes included) is unchanged across the treatment groups (F (2, 16) = 0.1, p = 0.9; Fig 8D). We show a significant effect of treatment on the CR+/DCX+/NeuN- and CR+/DCX+/NeuN+ phenotypes but not on the CR+/DCX-/NeuN- and CR+/DCX-/NeuN+ phenotypes (Table 1). Tg mice treated with *scyllo*-inositol/neotrofin combination had a lower percentage of CR+ cells that were CR+/DCX+/NeuN- compared to neotrofin, but not *scyllo*-inositol, treated animals (p = 0.003 and p = 0.41) and a higher percentage of CR+ cells that were CR+/DCX+/NeuN+ compared to neotrofin and *scyllo*-inositol treated mice (p = 0.0004 and p = 0.03, respectively; Table 1). Tg mice treated with *scyllo*-inositol also had a lower percentage of CR+ cells that were CR+/DCX+/NeuN- compared to neotrofin treated mice (p = 0.02) and trended to a higher percentage of CR+ cells that were CR+/DCX+/NeuN+ compared to the neotrofin treated mice (p = 0.06; Table 1). The chart in Fig 8E displays the combined mean percentages of all CR+ phenotypes, demonstrating the higher percentage of CR+/DCX+/NeuN+ and lower percentage of CR+/DCX+/NeuN- cells within *scyllo*-inositol/neotrofin combination treated mice compared to the other two treatment cohorts. We demonstrate that combination treated animals have a greater percentage of CR+/DCX+/NeuN+ cells, without a change in total CR+ cells. Overall, this data supports the conclusion that *scyllo*-inositol/neotrofin treatment is enhancing the differentiation/survival of neurons in the hippocampus.

**Table 1:** Phenotypic characterization of total CR cells in the subgranular zone and granular cell layer of the dentate gyrus in Tg mice treated with *scyllo*-inositol, neotrofin or *scyllo*-inositol/neotrofin.

Cell Type	Tg-SI mean (%) ± SEM (n = 6)	Tg-NEO mean (%) ± SEM (n = 6)	Tg-SI/NEO mean (%) ± SEM (n = 7)	F and p value^a^	Tg-SI vs Tg-NEO p value^a^	Tg-SI vs Tg-SI/NEO p value^a^	Tg-NEO vs Tg-SI/NEO p value^a^
**CR+/DCX-/NeuN-**	10.79 ± 1.77	10.95 ± 1.67	9.79 ± 1.58	F (2, 16) = 0.15, p = 0.86	N/A	N/A	N/A
**CR+/DCX+/NeuN-**	40.13 ± 4.24	51.22 ± 2.68	36.54 ± 2.06	F (2, 16) = 6.32, p = 0.0095	0.02	0.41	0.003
**CR+/DCX+/NeuN+**	27.83 ± 3.52	20.06 ± 2.11	36.38 ± 2.09	F (2, 16) = 10.06, p = 0.002	0.06	0.03	0.0004
**CR+/DCX-/NeuN+**	21.26 ± 6.06	17.77 ± 3.14	17.29 ± 2.36	F (2, 16) = 0.29, p = 0.76	N/A	N/A	N/A

CR, calretinin; DCX, doublecortin; NeuN, neuronal nuclei; Tg, TgCRND8; SI, *scyllo*-inositol; NEO, neotrofin.

^a^ F and p values were calculated using one-way ANOVA with Fisher’s Post-hoc test.

**Fig 9:**
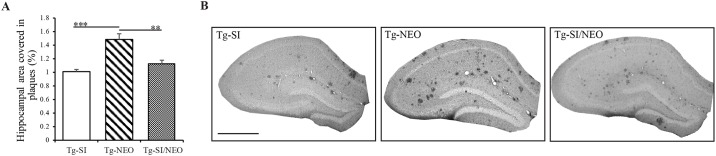
Hippocampal Aβ plaques in *scyllo*-inositol, neotrofin and *scyllo-*inositol/neotrofin treated 200 day old Tg mice. (A) Percent hippocampal area covered in plaques in 200 day old Tg-SI (n = 4), Tg-NEO (n = 4) and Tg-SI/NEO (n = 4) mice showed decreased plaques in Tg-SI and in Tg-SI/NEO compared to Tg-NEO. (B) Aβ plaques in the hippocampus of 200 day old mice demonstrate the reduction in plaques as a function of *scyllo*-inositol treatment. Scale bar indicates 200 μm. Data are mean ± SEM. One-way ANOVA with Fisher’s Post-hoc test, ** represents p< 0.01, *** represents p<0.001.

To ensure that *scyllo*-inositol was effective in the combination therapy, we quantified the hippocampal Aβ plaques after *scyllo*-inositol/neotrofin treatment in the 200 day old Tg mice. There was an overall effect of treatment on the level of Aβ pathology (F (2, 9) = 16.93, p = 0.0009; Fig 9A and 9B). *scyllo-*Inositol significantly reduced the percent hippocampal area covered in plaques by a third in both Tg-SI mice (p = 0.0003) and in Tg-SI/NEO mice (p = 0.002) compared to Tg-NEO mice (Fig 9A and 9B). There was no difference between Tg-SI and Tg-SI/NEO mice (p = 0.21).

## Discussion

To determine the role of Aβ-toxicity on adult hippocampal neurogenesis, we examined the effect of the Aβ-targeting compound, *scyllo*-inositol, in Tg mice at different stages of pathology. We demonstrate that in early AD-like pathology, reduction of Aβ is sufficient to restore neurogenesis to levels of NTg mice, however, in more established disease no effect was observed. As previously reported, long-term insult of Aβ induces changes in the neurogenic niche, specifically in neurotrophin signalling [13], which diminishes the effect of targeting solely Aβ. We therefore examined the effect of neotrofin alone, and in combination with *scyllo*-inositol, on adult hippocampal neurogenesis. We demonstrate that the combination treatment of *scyllo*-inositol/neotrofin enhances neuronal differentiation and survival.

In agreement with our results, J20 AD-mice have increased progenitor cell proliferation in young but not old mice [7] with corresponding data observed in AD patients [4]. Previous studies in TgCRND8 mice, however, have demonstrated contradictory results which may result from age- and methodological differences [8,31–33]. Furthermore, Aβ-targeted treatments, specifically passive immunization, are effective in promoting endogenous hippocampal neurogenesis in TgAD rodent models [34–35]. Similar to our results, enhanced neurogenesis in response to lithium treatment was observed in young but not aged TgCRND8 mice with a reduction in Aβ at both ages [32]. Although, the combination treatment of *scyllo*-inositol and neotrofin was not previously examined, *scyllo*-inositol has been tested in combination with R-flurbiprofen in TgAD mice. *scyllo-*inositol and R-flurbiprofen combination treated mice exhibited worsened AD-like pathology with treatment compared to *scyllo*-inositol alone [36]. Recently, other combination therapeutic strategies that targeted neurogenesis in AD mouse models showed no beneficial outcomes [37–38].

Firstly, we examined the effect of *scyllo*-inositol treatment on hippocampal neurogenesis in early AD-like phenotype exhibiting low Aβ and in late AD-like phenotype with significant Aβ accumulation [26]. As previously reported, *scyllo-*inositol decreased Aβ levels within the hippocampus at both stages of disease [18]. In young TgCRND8 mice, the increase in NPC proliferation we observed may represent a compensatory response to decreased survival of newborn cells. *scyllo*-Inositol attenuated this compensatory proliferation and vastly increased survival. Thus, we propose that *scyllo*-inositol decreased Aβ-injury directly to NPCs, and increased negative feedback on NPC proliferation. Our results are supported by the demonstration that TgCRND8-derived NPCs release Aβ and exhibit decreased viability [8] and restriction of Aβ production to mature neurons *in vivo* had no effect on neurogenesis [39]. Furthermore, studies have shown that the neurogenic niche is intact early in Aβ-deposition [13,40]. *scyllo*-Inositol did not increase survival in NTg mice, further suggesting that the increased survival rate is likely the result of removal of Aβ.

*scyllo*-Inositol specifically rescued cell proliferation and survival deficits in TgCRND8 mice to those of NTgs. Increasing the number of proliferating and, more importantly surviving neurons is critical in many hippocampal-dependent behaviours, including pattern separation [9]. The importance and integration of surviving neurons in these behaviours is a complex process [reviewed in 9,41–42]. Interventions to increase newborn granule cells in adults improves spatial and contextual discrimination [10,43], while ablating neurogenesis impairs discrimination behaviours [44–45]. Thus *scyllo-*inositol-induced memory benefits in TgCRND8 mice [18] may in part result from enhanced production and survival of newborn neurons in the hippocampus.

In older TgCRND8 mice with late AD-like phenotype decreased proliferating NPCs with no change in survival was observed. The percent of neuronal proliferating cells was similar to NTgs, however TgCRND8 mice had decreased neuronal survival regardless of *scyllo-*Inositol treatment. In aging and AD, dysfunction within the neurogenic niche will contribute to reduction in NPC proliferation, maturation and survival of new neurons [46–47]. Additionally, a population of BrdU+ cells counted were not co-labelled with the markers we stained for. Analysis of the phenotype of BrdU+ cells that were not DCX+, NeuN+ or GFAP+, however, was outside the scope of our work. We hypothesize, that this population of cells may be made up of microglia or endothelial cells that during replication incorporated BrdU into their DNA.

Although *scyllo*-inositol reduced Aβ burden, the overexpression of APP remains unaffected by treatment and may contribute to deficits in neurogenesis. Knockout of APP in GABAergic neurons, notably in interneurons of the dentate gyrus, impairs GABAergic firing and hippocampal neurogenesis [48], indicating the role of regulated levels of endogenous APP on neurogenesis. Therefore, prolonged overexpression of APP in the aged TgCRND8 mice may contribute to impairment in the neurogenic niche. In support of this, TgCRND8 mice have decreased hippocampal GABAergic interneurons compared to NTg mice [40]. These data suggest that in late AD combination therapeutic approaches are necessary to support the neurogenic niche.

The neurotrophin environment, known to regulate neurogenesis through cell survival, differentiation and apoptotic signaling, is affected in AD [17]. A loss of BDNF transcript levels was demonstrated in 6–8 month old TgCRND8 mice [13]. AβPP/PS1 mice crossed with BDNF+/- mice exhibited decreased DCX+ cell density compared to those crossed with BDNF+/+ mice [49]. BDNF has the strongest affinity for the TrkB receptor, which is expressed on hippocampal NPCs [50], suggesting a direct effect of BDNF on proliferating cells in the dentate gyrus. Furthermore, decreases in mature NGF and its receptor, and an increase in pro-NGF, have been reported in AD and AD models [51–53]. Thus survival and maturation of hippocampal NPCs may be compromised by deficits in neurotrophins during disease progression.

Administration of BDNF and NGF have been investigated as treatments for AD, however invasive delivery methods diminish ease of translation [54]. Therefore, we examined a combined treatment of reducing Aβ levels with *scyllo-*inositol and induction of neurotrophins with neotrofin, both readily cross the BBB [19,23]. We hypothesized that *scyllo-*inositol and neotrofin combined treatment would increase the number of hippocampal proliferating cells and the percentage of neurons, however we observed no differences in either measure. In support of this, NTg mice which received a single dose of neotrofin demonstrated increased hippocampal proliferation, however, mice who received multiple doses, similar to the TgCRND8 mice in this study, had no change in cell proliferation [25,55]. These data in combination with our results suggest that multiple dosages of neotrofin exerts an effect at a later stage of hippocampal neurogenesis. We hypothesized that treatment with *scyllo*-inositol/neotrofin would lead to a higher percentage of neuroblasts and immature neurons (DCX+ cells) that differentiate into DCX+/CR+ cells, but determined no statistical differences between groups. The DCX+/CR+ population of cells are immature granule cells at the transition between exiting the cell cycle and full neuronal maturation. Survival of neurons at 2 weeks after birth represented cells that express DCX and CR [56], and was indicative of long-term cell survival [57–59]. Since TgCRND8 mice with advanced AD-like pathology have impaired neuronal survival, we hypothesized that *scyllo*-inositol/neotrofin combination treatment may enhance survival of these immature neurons.

In support of this hypothesis, we demonstrate that the percent of late-stage immature neurons (DCX+/CR+/NeuN+ cells) is highest in the *scyllo*-inositol/neotrofin combination treated mice compared to the other two treatment cohorts. Of the DCX+/CR+ cells in the combination treatment cohort 49.89% (± 1.18) were also NeuN+, which was greater than 40.62% (± 1.35) and 27.99% (± 1.96) in the *scyllo*-inositol and neotrofin treated mice, respectively. It is interesting to note that the neotrofin treatment cohort had a percentage of DCX cells that were immature neurons (DCX+/CR+) comparable to the other two cohorts, however fewer cells continue to mature and express NeuN. The majority of newborn neurons are lost between 2 and 3 weeks post-birth, which is approximately when immature neurons are approaching a degree of maturity characterized by NeuN expression [60]. Continual Aβ production from these immature neurons may contribute to the reduction in viability of these cells, indicating the importance of combining this treatment with *scyllo*-inositol. There was no difference in total DCX+ cells between *scyllo-*inositol, neotrofin and combination treatments. These results demonstrate that *scyllo-*inositol/neotrofin treatment promotes differentiation of DCX+ neurons into immature neurons, with no effects on proliferation. This pro-differentiation effect may positively feedback to stabilize existing neuroblasts, thus pushing the maturity of the developing neurons forward. In agreement with our results, non-transgenic rats infused with recombinant human NGF had increased hippocampal cell survival at 2 weeks post-BrdU injection with no changes in proliferation [57]. Additionally, we demonstrate that a similar shift towards an increased percentage of immature neurons expressing CR+/DCX+/NeuN+ and a decreased percentage of expressing CR+/DCX+/NeuN- within the dentate gyrus CR cell population, in *scyllo*-inositol/neotrofin treatment compared to both single treatments, and in *scyllo*-inositol treatment compared to neotrofin treatment.

This data supports the conclusion that the combination treatment enhances neuronal differentiation/survival. We hypothesize the beneficial mechanism of action in the combination treated TgCRND8 mice occurs through neotrofin treatment promoting the health of the neurogenic environment thereby increasing cell differentiation/survival, and *scyllo*-inositol treatment reducing continual Aβ insult, which helps stabilize newborn cells.

In AD there is a decline in healthy endogenous neurogenesis, with high variability in the observed effect between humans, animal models and age [46]. We demonstrate that TgCRND8 mice with early AD-like pathology exhibit increased progenitor cell proliferation with decreased neuronal phenotype. Removal of Aβ by *scyllo*-inositol rescued these deficits and increased the number of surviving cells. However, *scyllo*-inositol had no effect on neurogenesis in late AD-like pathology. We found that targeting only Aβ at this age was insufficient to rescue neurogenesis. We demonstrate that *scyllo*-inositol/neotrofin combination treatment increased the percentage of neurons surviving and differentiating. s*cyllo*-Inositol and neotrofin together exert a beneficial effect on the maturation stage of neurogenesis that may have significance for improved memory and cognition. This combined treatment paradigm of targeting Aβ and inducing neurotrophins is a proof of concept for AD preclinical multi-drug investigations and warrants further investigation.

## Supporting Information

S1 Fig Raw data for all results. (XLSX)
